# Bis[4-(2-hydroxy­benzyl­ideneamino)benzoato-κ*O*]tetrakis­(methanol-κ*O*)manganese(II)

**DOI:** 10.1107/S160053680901681X

**Published:** 2009-05-14

**Authors:** Min Zhong, Jia-Huang Lin, Jing Shang, Ting-Hong Huang, Xiu-Jian Wang

**Affiliations:** a School of Chemistry and Chemical Engineering, Guangxi Normal University, Guilin 541004, People’s Republic of China

## Abstract

In the title mononuclear complex, [Mn(C_14_H_10_NO_3_)_2_(CH_3_OH)_4_], the Mn^II^ atom, lying on an inversion centre, exhibits a distorted octa­hedral geometry, defined by two O atoms from two monodentate ligands and four O atoms from four methanol mol­ecules. The crystal structure involves intra­molecular O—H⋯N and O—H⋯O and inter­molecular O—H⋯O hydrogen bonds.

## Related literature

For general background, see: Deeth (2008 ▶); Dubois *et al.* (2008 ▶); Huang *et al.* (2004 ▶).
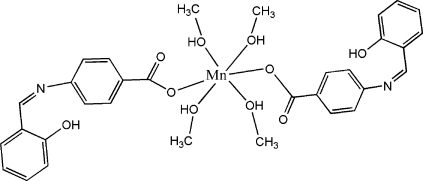


## Experimental

### 

#### Crystal data

[Mn(C_14_H_10_NO_3_)_2_(CH_4_O)_4_]
*M* *_r_* = 663.57
Monoclinic, 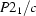

*a* = 15.0341 (6) Å
*b* = 11.8819 (4) Å
*c* = 8.8178 (3) Å
β = 98.912 (4)°
*V* = 1556.14 (10) Å^3^
*Z* = 2
Mo *K*α radiation
μ = 0.49 mm^−1^
*T* = 293 K
0.6 × 0.6 × 0.3 mm

#### Data collection

Oxford Diffraction Gemini S Ultra diffractometer
Absorption correction: multi-scan (*CrysAlis RED*; Oxford Diffraction, 2006 ▶) *T*_min_ = 0.823, *T*_max_ = 1.000 (expected range = 0.711–0.865)
10167 measured reflections
3374 independent reflections
2008 reflections with *I* > 2σ(*I*)
*R*_int_ = 0.032

#### Refinement

*R*[*F* ^2^ > 2σ(*F* ^2^)] = 0.039
*wR*(*F* ^2^) = 0.102
*S* = 0.92
3374 reflections
205 parameters
H-atom parameters constrained
Δρ_max_ = 0.55 e Å^−3^
Δρ_min_ = −0.31 e Å^−3^

### 

Data collection: *CrysAlis CCD* (Oxford Diffraction, 2006 ▶); cell refinement: *CrysAlis RED* (Oxford Diffraction, 2006 ▶); data reduction: *CrysAlis RED*; program(s) used to solve structure: *SHELXS97* (Sheldrick, 2008 ▶); program(s) used to refine structure: *SHELXL97* (Sheldrick, 2008 ▶); molecular graphics: *SHELXTL* (Sheldrick, 2008 ▶) and *Mercury* (Macrae *et al.*, 2006 ▶); software used to prepare material for publication: *SHELXTL*.

## Supplementary Material

Crystal structure: contains datablocks I, New_Global_Publ_Block. DOI: 10.1107/S160053680901681X/hy2192sup1.cif

Structure factors: contains datablocks I. DOI: 10.1107/S160053680901681X/hy2192Isup2.hkl

Additional supplementary materials:  crystallographic information; 3D view; checkCIF report

## Figures and Tables

**Table 1:** Selected bond lengths (Å)

Mn1—O3	2.1275 (15)
Mn1—O5	2.1803 (13)
Mn1—O4	2.2023 (14)

**Table 2:** Hydrogen-bond geometry (Å, °)

*D*—H⋯*A*	*D*—H	H⋯*A*	*D*⋯*A*	*D*—H⋯*A*
O1—H1*A*⋯N1	0.85	1.83	2.619 (2)	153
O4—H4*B*⋯O2^i^	0.85	1.84	2.621 (2)	151
O5—H5*B*⋯O2	0.85	1.83	2.618 (2)	153

Symmetry code: (i) 
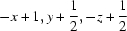
.

**Fig. 1.**
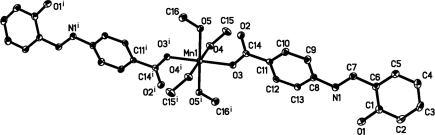
Molecular structure of the title compound. Displacement ellipsoids are drawn at the 30% probability level. H atoms have been omitted for clarity. [Symmetry code: (i) -x + 1, -y, -z.]

**Fig. 2.**
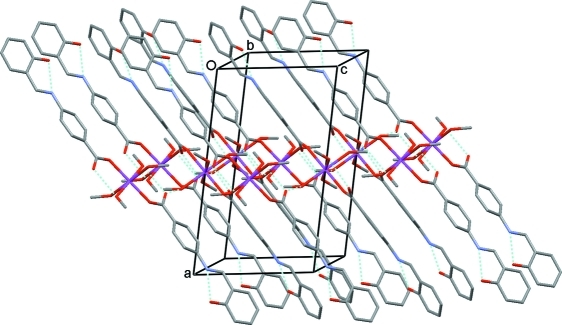
Crystal packing of the title compound, showing hydrogen bonds (dashed lines).

## supplementary crystallographic information

### Comment

General molecular mechanics method for transition metal carboxylates and the multiple coordination modes in manganese(II) complexes have been reported recently (Deeth, 2008). Information on the structures of manganese(II) carboxylates continues to be collected, and at the same time new applications of such complexes are being discovered in magnetic properties, potential biological significance and ferrimagnet (Huang *et al.*, 2004). The chemistry of organo-manganese(II) complexes of Schiff base has stemmed from the reported biocidal and catalytic activities of organo-manganese(II) compounds (Dubois *et al.*, 2008). We report here a new monomeric manganese(II) compound, which contains the Schiff base ligand, *N*-(4-carboxyphenyl)salicylideneimine (Fig.1). The Mn^II^ atom has a distorted octahedral geometry (Table 1). There exist intra- and intermolecular hydrogen bonds in the crystal structure (Table 2). The intermolecular hydrogen bonds is used to form a two-dimensional supramolecular network (Fig. 2).

### Experimental

Manganese(II) acetate tetrahydrate (0.049 g, 0.2 mmol) was dissolved in 8 ml deionized water, giving a transparent solution (A), and *N*-(4-carboxyphenyl)salicylideneimine (0.097 g, 0.4 mmol) was dissolved in 10 ml me thanol (B). Then solution B was mixed with A and a suspension was obtained. Ammonia was added to the above mixture dropwise under magnetic stirring until pH value is neutral. The resulting suspension was transferred into a 25 ml Teflon-lined stainless-steel autoclave. The autoclave was sealed and maintained at 363 K for 12 h under autogenous pressure. After the reaction was completed, the resulting colourless block crystals were collected by filtration.

### Refinement

H atoms were positioned geometrically and refined as riding atoms, with C—H = 0.93 (aromatic) and 0.96 (CH_3_) Å and O—H = 0.85 Å, and with *U*_iso_(H) = 1.2*U*_eq_(C,*O*).

### Crystal data

**Table:**

[Mn(C_14_H_10_NO_3_)_2_(CH_4_O)_4_]	*F*(000) = 694
*M**_r_* = 663.57	*D*_x_ = 1.416 Mg m^−^^3^
Monoclinic, *P*2_1_/*c*	Mo *K*α radiation, λ = 0.71073 Å
Hall symbol: -P 2ybc	Cell parameters from 3622 reflections
*a* = 15.0341 (6) Å	θ = 2.7–29.8°
*b* = 11.8819 (4) Å	µ = 0.49 mm^−^^1^
*c* = 8.8178 (3) Å	*T* = 293 K
β = 98.912 (4)°	Block, colorless
*V* = 1556.14 (10) Å^3^	0.6 × 0.6 × 0.3 mm
*Z* = 2	

### Data collection

**Table:**

Oxford Diffraction Gemini S Ultra diffractometer	3374 independent reflections
Radiation source: Enhance (Mo) X-ray Source	2008 reflections with *I* > 2σ(*I*)
graphite	*R*_int_ = 0.032
Detector resolution: 16.0855 pixels mm^-1^	θ_max_ = 27.0°, θ_min_ = 2.7°
ω scans	*h* = −19→16
Absorption correction: multi-scan (*CrysAlis RED*; Oxford Diffraction, 2006)	*k* = −15→14
*T*_min_ = 0.823, *T*_max_ = 1.000	*l* = −11→11
10167 measured reflections	

### Refinement

**Table:**

Refinement on *F*^2^	Primary atom site location: structure-invariant direct methods
Least-squares matrix: full	Secondary atom site location: difference Fourier map
*R*[*F*^2^ > 2σ(*F*^2^)] = 0.039	Hydrogen site location: inferred from neighbouring sites
*wR*(*F*^2^) = 0.102	H-atom parameters constrained
*S* = 0.92	*w* = 1/[σ^2^(*F*_o_^2^) + (0.058*P*)^2^] where *P* = (*F*_o_^2^ + 2*F*_c_^2^)/3
3374 reflections	(Δ/σ)_max_ < 0.001
205 parameters	Δρ_max_ = 0.55 e Å^−^^3^
0 restraints	Δρ_min_ = −0.31 e Å^−^^3^

### Fractional atomic coordinates and isotropic or equivalent isotropic displacement parameters (Å^2^)

**Table:**

	*x*	*y*	*z*	*U*_iso_*/*U*_eq_	
Mn1	0.5000	0.0000	0.0000	0.02171 (15)	
O1	1.09324 (11)	0.03911 (14)	0.80222 (19)	0.0404 (4)	
H1A	1.0420	0.0210	0.7533	0.048*	
O2	0.57922 (10)	−0.20216 (13)	0.26345 (18)	0.0350 (4)	
O3	0.60735 (10)	−0.03061 (12)	0.18268 (17)	0.0291 (4)	
N1	0.94227 (12)	−0.06941 (15)	0.7155 (2)	0.0295 (5)	
C1	1.11213 (15)	−0.0410 (2)	0.9117 (3)	0.0303 (6)	
C2	1.19528 (16)	−0.0391 (2)	1.0049 (3)	0.0360 (6)	
H2A	1.2378	0.0151	0.9904	0.043*	
C3	1.21437 (16)	−0.1187 (2)	1.1196 (3)	0.0397 (6)	
H3A	1.2704	−0.1177	1.1815	0.048*	
C4	1.15283 (15)	−0.1989 (2)	1.1442 (3)	0.0377 (6)	
H4A	1.1665	−0.2511	1.2229	0.045*	
C5	1.07051 (15)	−0.2014 (2)	1.0510 (3)	0.0332 (6)	
H5A	1.0288	−0.2561	1.0675	0.040*	
C6	1.04831 (14)	−0.12450 (18)	0.9335 (2)	0.0276 (5)	
C7	0.96278 (15)	−0.13309 (19)	0.8321 (3)	0.0306 (5)	
H7A	0.9215	−0.1870	0.8529	0.037*	
C8	0.86185 (14)	−0.08573 (19)	0.6112 (2)	0.0269 (5)	
C9	0.81878 (14)	−0.18938 (19)	0.5844 (3)	0.0312 (6)	
H9A	0.8412	−0.2524	0.6402	0.037*	
C10	0.74265 (14)	−0.19842 (19)	0.4748 (3)	0.0299 (5)	
H10A	0.7137	−0.2675	0.4587	0.036*	
C11	0.70884 (14)	−0.10594 (17)	0.3887 (2)	0.0225 (5)	
C12	0.75350 (15)	−0.00380 (18)	0.4146 (2)	0.0259 (5)	
H12A	0.7322	0.0587	0.3567	0.031*	
C13	0.82848 (15)	0.00641 (19)	0.5241 (2)	0.0279 (5)	
H13A	0.8572	0.0757	0.5401	0.033*	
C14	0.62646 (14)	−0.11436 (18)	0.2699 (2)	0.0234 (5)	
O4	0.42496 (10)	0.08439 (12)	0.16313 (16)	0.0306 (4)	
H4B	0.4371	0.1543	0.1666	0.037*	
O5	0.43893 (10)	−0.15779 (11)	0.05698 (16)	0.0271 (4)	
H5B	0.4791	−0.1938	0.1163	0.033*	
C15	0.42208 (19)	0.0436 (2)	0.3134 (3)	0.0436 (7)	
H15A	0.3862	0.0932	0.3650	0.065*	
H15B	0.4821	0.0403	0.3695	0.065*	
H15C	0.3960	−0.0303	0.3075	0.065*	
C16	0.39223 (16)	−0.23800 (19)	−0.0468 (3)	0.0368 (6)	
H16A	0.3730	−0.2999	0.0103	0.055*	
H16B	0.4316	−0.2651	−0.1145	0.055*	
H16C	0.3406	−0.2029	−0.1058	0.055*	

### Atomic displacement parameters (Å^2^)

**Table:**

	*U*^11^	*U*^22^	*U*^33^	*U*^12^	*U*^13^	*U*^23^
Mn1	0.0265 (3)	0.0162 (2)	0.0218 (3)	−0.0006 (2)	0.00172 (19)	0.0002 (2)
O1	0.0353 (10)	0.0405 (10)	0.0447 (11)	−0.0090 (8)	0.0041 (8)	0.0001 (9)
O2	0.0365 (9)	0.0240 (9)	0.0407 (10)	−0.0064 (7)	−0.0061 (8)	0.0091 (8)
O3	0.0313 (9)	0.0259 (9)	0.0282 (9)	−0.0022 (7)	−0.0011 (7)	0.0067 (7)
N1	0.0296 (11)	0.0303 (11)	0.0282 (10)	−0.0001 (9)	0.0029 (9)	−0.0037 (9)
C1	0.0333 (14)	0.0307 (13)	0.0272 (12)	0.0057 (10)	0.0061 (11)	−0.0047 (11)
C2	0.0260 (13)	0.0373 (14)	0.0454 (15)	−0.0033 (11)	0.0077 (12)	−0.0122 (12)
C3	0.0247 (14)	0.0523 (17)	0.0392 (15)	0.0064 (12)	−0.0040 (11)	−0.0083 (13)
C4	0.0366 (14)	0.0417 (15)	0.0331 (14)	0.0070 (12)	0.0002 (12)	0.0001 (12)
C5	0.0286 (13)	0.0352 (14)	0.0354 (14)	−0.0022 (11)	0.0035 (11)	−0.0022 (12)
C6	0.0223 (12)	0.0336 (13)	0.0271 (12)	0.0010 (10)	0.0044 (10)	−0.0069 (11)
C7	0.0304 (13)	0.0313 (13)	0.0302 (13)	−0.0033 (10)	0.0053 (11)	−0.0027 (11)
C8	0.0245 (12)	0.0312 (13)	0.0249 (12)	−0.0004 (10)	0.0036 (10)	−0.0059 (10)
C9	0.0293 (13)	0.0247 (13)	0.0373 (14)	0.0055 (10)	−0.0015 (11)	0.0034 (11)
C10	0.0293 (13)	0.0226 (12)	0.0355 (14)	−0.0011 (10)	−0.0022 (11)	−0.0010 (11)
C11	0.0252 (12)	0.0214 (11)	0.0225 (11)	0.0003 (9)	0.0086 (10)	−0.0014 (9)
C12	0.0330 (12)	0.0224 (11)	0.0223 (11)	0.0000 (11)	0.0043 (9)	0.0032 (10)
C13	0.0336 (13)	0.0244 (12)	0.0251 (11)	−0.0048 (11)	0.0025 (10)	−0.0020 (11)
C14	0.0276 (12)	0.0201 (12)	0.0237 (11)	0.0025 (10)	0.0075 (10)	−0.0001 (10)
O4	0.0433 (10)	0.0183 (8)	0.0317 (9)	0.0003 (7)	0.0105 (7)	−0.0017 (7)
O5	0.0306 (8)	0.0180 (8)	0.0309 (9)	−0.0023 (7)	−0.0010 (7)	0.0019 (7)
C15	0.070 (2)	0.0313 (13)	0.0339 (14)	−0.0014 (13)	0.0207 (14)	0.0006 (12)
C16	0.0417 (15)	0.0264 (13)	0.0415 (15)	−0.0077 (11)	0.0041 (12)	−0.0096 (11)

### Geometric parameters (Å, °)

**Table:**

Mn1—O3	2.1275 (15)	C7—H7A	0.9300
Mn1—O3^i^	2.1275 (15)	C8—C13	1.386 (3)
Mn1—O5^i^	2.1802 (13)	C8—C9	1.394 (3)
Mn1—O5	2.1803 (13)	C9—C10	1.383 (3)
Mn1—O4	2.2023 (14)	C9—H9A	0.9300
Mn1—O4^i^	2.2023 (14)	C10—C11	1.387 (3)
O1—C1	1.354 (3)	C10—H10A	0.9300
O1—H1A	0.8500	C11—C12	1.389 (3)
O2—C14	1.258 (2)	C11—C14	1.496 (3)
O3—C14	1.263 (2)	C12—C13	1.372 (3)
N1—C7	1.275 (3)	C12—H12A	0.9300
N1—C8	1.415 (3)	C13—H13A	0.9300
C1—C2	1.386 (3)	O4—C15	1.418 (3)
C1—C6	1.414 (3)	O4—H4B	0.8500
C2—C3	1.382 (3)	O5—C16	1.428 (2)
C2—H2A	0.9300	O5—H5B	0.8500
C3—C4	1.369 (3)	C15—H15A	0.9600
C3—H3A	0.9300	C15—H15B	0.9600
C4—C5	1.376 (3)	C15—H15C	0.9600
C4—H4A	0.9300	C16—H16A	0.9600
C5—C6	1.383 (3)	C16—H16B	0.9600
C5—H5A	0.9300	C16—H16C	0.9600
C6—C7	1.452 (3)		
			
O3—Mn1—O3^i^	180.00 (10)	C13—C8—C9	119.0 (2)
O3—Mn1—O5^i^	91.39 (5)	C13—C8—N1	116.85 (19)
O3^i^—Mn1—O5^i^	88.61 (5)	C9—C8—N1	124.0 (2)
O3—Mn1—O5	88.61 (5)	C10—C9—C8	120.0 (2)
O3^i^—Mn1—O5	91.39 (5)	C10—C9—H9A	120.0
O5^i^—Mn1—O5	180.00 (7)	C8—C9—H9A	120.0
O3—Mn1—O4	89.34 (6)	C9—C10—C11	121.0 (2)
O3^i^—Mn1—O4	90.66 (6)	C9—C10—H10A	119.5
O5^i^—Mn1—O4	92.04 (5)	C11—C10—H10A	119.5
O5—Mn1—O4	87.96 (5)	C10—C11—C12	118.4 (2)
O3—Mn1—O4^i^	90.66 (6)	C10—C11—C14	121.60 (19)
O3^i^—Mn1—O4^i^	89.34 (6)	C12—C11—C14	120.02 (19)
O5^i^—Mn1—O4^i^	87.96 (5)	C13—C12—C11	121.1 (2)
O5—Mn1—O4^i^	92.04 (5)	C13—C12—H12A	119.4
O4—Mn1—O4^i^	180.00 (7)	C11—C12—H12A	119.4
C1—O1—H1A	104.9	C12—C13—C8	120.5 (2)
C14—O3—Mn1	132.05 (14)	C12—C13—H13A	119.7
C7—N1—C8	121.23 (19)	C8—C13—H13A	119.7
O1—C1—C2	118.8 (2)	O2—C14—O3	123.5 (2)
O1—C1—C6	121.2 (2)	O2—C14—C11	119.14 (19)
C2—C1—C6	120.0 (2)	O3—C14—C11	117.32 (18)
C3—C2—C1	119.3 (2)	C15—O4—Mn1	123.20 (14)
C3—C2—H2A	120.4	C15—O4—H4B	109.7
C1—C2—H2A	120.4	Mn1—O4—H4B	109.9
C4—C3—C2	121.6 (2)	C16—O5—Mn1	127.52 (13)
C4—C3—H3A	119.2	C16—O5—H5B	106.9
C2—C3—H3A	119.2	Mn1—O5—H5B	106.9
C3—C4—C5	119.2 (2)	O4—C15—H15A	109.5
C3—C4—H4A	120.4	O4—C15—H15B	109.5
C5—C4—H4A	120.4	H15A—C15—H15B	109.5
C4—C5—C6	121.6 (2)	O4—C15—H15C	109.5
C4—C5—H5A	119.2	H15A—C15—H15C	109.5
C6—C5—H5A	119.2	H15B—C15—H15C	109.5
C5—C6—C1	118.4 (2)	O5—C16—H16A	109.5
C5—C6—C7	120.2 (2)	O5—C16—H16B	109.5
C1—C6—C7	121.4 (2)	H16A—C16—H16B	109.5
N1—C7—C6	122.4 (2)	O5—C16—H16C	109.5
N1—C7—H7A	118.8	H16A—C16—H16C	109.5
C6—C7—H7A	118.8	H16B—C16—H16C	109.5
			
O5^i^—Mn1—O3—C14	−173.33 (18)	C8—C9—C10—C11	1.0 (3)
O5—Mn1—O3—C14	6.67 (18)	C9—C10—C11—C12	0.3 (3)
O4—Mn1—O3—C14	94.64 (19)	C9—C10—C11—C14	−179.90 (18)
O4^i^—Mn1—O3—C14	−85.36 (19)	C10—C11—C12—C13	−1.0 (3)
O1—C1—C2—C3	178.3 (2)	C14—C11—C12—C13	179.17 (18)
C6—C1—C2—C3	−0.6 (3)	C11—C12—C13—C8	0.4 (3)
C1—C2—C3—C4	−0.5 (4)	C9—C8—C13—C12	0.8 (3)
C2—C3—C4—C5	1.0 (4)	N1—C8—C13—C12	176.55 (18)
C3—C4—C5—C6	−0.2 (3)	Mn1—O3—C14—O2	−1.2 (3)
C4—C5—C6—C1	−0.9 (3)	Mn1—O3—C14—C11	−179.75 (12)
C4—C5—C6—C7	176.7 (2)	C10—C11—C14—O2	10.7 (3)
O1—C1—C6—C5	−177.6 (2)	C12—C11—C14—O2	−169.4 (2)
C2—C1—C6—C5	1.3 (3)	C10—C11—C14—O3	−170.6 (2)
O1—C1—C6—C7	4.8 (3)	C12—C11—C14—O3	9.2 (3)
C2—C1—C6—C7	−176.2 (2)	O3—Mn1—O4—C15	−41.12 (17)
C8—N1—C7—C6	174.53 (19)	O3^i^—Mn1—O4—C15	138.88 (17)
C5—C6—C7—N1	−174.7 (2)	O5^i^—Mn1—O4—C15	−132.49 (17)
C1—C6—C7—N1	2.8 (3)	O5—Mn1—O4—C15	47.51 (17)
C7—N1—C8—C13	157.1 (2)	O3—Mn1—O5—C16	−142.12 (16)
C7—N1—C8—C9	−27.4 (3)	O3^i^—Mn1—O5—C16	37.88 (16)
C13—C8—C9—C10	−1.5 (3)	O4—Mn1—O5—C16	128.49 (16)
N1—C8—C9—C10	−176.92 (19)	O4^i^—Mn1—O5—C16	−51.51 (16)

Symmetry codes: (i) −*x*+1, −*y*, −*z*.

### Hydrogen-bond geometry (Å, °)

**Table:**

*D*—H···*A*	*D*—H	H···*A*	*D*···*A*	*D*—H···*A*
O1—H1A···N1	0.85	1.83	2.619 (2)	153
O4—H4B···O2^ii^	0.85	1.84	2.621 (2)	151
O5—H5B···O2	0.85	1.83	2.618 (2)	153

Symmetry codes: (ii) −*x*+1, *y*+1/2, −*z*+1/2.
